# Induction of endoplasmic reticulum stress is associated with the anti‐tumor activity of monepantel across cancer types

**DOI:** 10.1002/cam4.6021

**Published:** 2023-05-06

**Authors:** Tiffany J. Harris, Yang Liao, Wei Shi, Marco Evangelista, Bhupinder Pal, Hamsa Puthalakath, Roger Aston, Richard Mollard, John M. Mariadason, Erinna F. Lee, Walter D. Fairlie

**Affiliations:** ^1^ Olivia Newton‐John Cancer Research Institute Heidelberg Victoria Australia; ^2^ School of Cancer Medicine La Trobe University Bundoora Victoria Australia; ^3^ Department of Biochemistry and Chemistry, School of Agriculture, Biomedicine and Environment, La Trobe Institute for Molecular Science La Trobe University Bundoora Victoria Australia; ^4^ PharmAust Ltd Claremont Australia; ^5^ Faculty of Veterinary and Agricultural Sciences University of Melbourne Parkville Victoria Australia

**Keywords:** autophagy, cell cycle, ER stress, monepantel, mTOR

## Abstract

**Background:**

Monepantel is an anti‐helminthic drug that also has anti‐cancer properties. Despite several studies over the years, the molecular target of monepantel in mammalian cells is still unknown, and its mechanism‐of‐action is not fully understood, though effects on cell cycle, mTOR signalling and autophagy have been implicated.

**Methods:**

Viability assays were performed on >20 solid cancer cell cells, and apoptosis assays were performed on a subset of these, including 3D cultures. Genetic deletion of BAX/BAK and ATG were used to establish roles of apoptosis and autophagy in killing activity. RNA‐sequencing was performed on four cell lines after monepantel treatment, and differentially regulated genes were confirmed by Western blotting.

**Results:**

We showed that monepantel has anti‐proliferative activity on a broad range of cancer cell lines. In some, this was associated with induction of apoptosis which was confirmed using a BAX/BAK‐deficient cell line. However, proliferation is still inhibited in these cells following monepantel treatment, indicating cell‐cycle disruption as the major anti‐cancer effect. Previous studies have also indicated autophagic cell death occurs following monepantel treatment. We showed autophagy induction in multiple cell lines; however, deletion of a key autophagy regulator ATG7 had minimal impact on monepantel’s anti‐proliferative activity, suggesting autophagy is associated with, but not required for its anti‐tumour effects. Transcriptomic analysis of four cell lines treated with monepantel revealed downregulation of many genes involved in the cell cycle, and upregulation of genes linked to ATF4‐mediated ER stress responses, especially those involved in amino‐acid metabolism and protein synthesis.

**Conclusions:**

As these outcomes are all associated with mTOR signalling, cell cycle and autophagy, we now provide a likely triggering mechanism for the anti‐cancer activity of monepantel.

## 1 INTRODUCTION

Monepantel was the first of a new (“amino‐acetonitrile”) class of broad‐acting anti‐helminthic agents used to treat nematode infections in livestock including sheep and cattle.^1^ Its primary mode‐of‐action in nematodes is by acting as an allosteric agonist of the DEG‐3 subtype of nicotinic acetylcholine receptors, causing muscle hypercontraction leading to spastic paralysis and death.^2^, ^3^, ^4^, ^5^ The excellent safety profile of the drug likely reflects that these receptors are not conserved outside of nematodes,^1^ resulting in it being well‐tolerated in mammals^1^, ^6^ with recent studies showing it does not possess genotoxic or carcinogenic properties in species such as mice, rats, and dogs.^7^

Like other anti‐helminthic agents (e.g., ivermectin, levamisole, albendazole),^8^ monepantel has been shown to also possess anti‐tumor activity.^9^, ^10^, ^11^, ^12^ To date, this has primarily been demonstrated in studies on ovarian cancer using cell lines in vitro where its activity is typically in the EC_50_ 10–30 μM range, and in vivo using xenograft models.^9^, ^10^, ^11^, ^12^ It can also act synergistically with other anti‐cancer drugs such as doxorubicin.^9^ These studies have mostly linked the anti‐tumor activity of monepantel to the induction of autophagy through the mTOR/p70S6K/4EBP1 axis and inhibition of cell cycle progression due to downregulation of cyclins A, D1 and E2, and Cdk2/4 and upregulation of p27.^10^, ^11^, ^12^ Coupled with its well‐understood safety profile, these data have prompted at least one Phase I clinical trial of monepantel in humans performed on late‐stage patients where other standard treatments for a range of cancers (colorectal, small‐cell, and non‐small cell lung cancer) have failed.^13^ This small study provided useful pharmacokinetic data and some evidence of anti‐cancer activity. However, it also revealed issues such as with poor palatability,^13^ though recent studies in dogs suggest these have now been resolved.

Beyond ovarian cancer, there are very little data on whether monepantel is effective in other cancers. We recently reported similar (micromolar) activity to that seen in ovarian cancer cell lines on a small number of cell lines reflecting the cancer types studied in the Phase I trial, though this was not wide‐ranging.^13^ Most mechanism‐of‐action studies of monepantel to date have taken candidate‐based approaches; however, it is still not clear whether autophagy induction is required for monepantel cell killing or just associated with it, and whether this is an ovarian cancer‐specific mechanism. In this paper, we assessed monepantel activity in a significantly greater number of cancer types and cell lines than previously studied and showed broad, cancer‐agnostic cell‐killing. Moreover, we have used an unbiased RNA‐sequencing approach to determine monepantel's mechanism‐of‐action, providing important new information on potential pathways involved that could be exploited in future studies.

## 2 MATERIALS AND METHODS

### 2.1 Drugs and cell lines

Monepantel was provided by PharmAust and a stock solution prepared at 10 mM in 100% ethanol and stored at −20°C. Vorinostat was purchased from Selleck (Cat#A1047) and 5FU from Sigma (Cat#F6627‐1G). Both were prepared as 10 mM stock solutions in phosphate‐buffered saline (5FU) or DMSO (Vorinostat). Cell lines were originally obtained from ATCC except HCT 116 which was gift from Associate Professor Grant Dewson (WEHI) with identities confirmed by STR profiling and shown to be mycoplasma negative based on in‐house MycoAlert assays (Lonza). All were maintained at 37°C with 10% CO_2_ except U‐87 MG and SW620 which were grown in 5% CO_2_. Cell lines were cultured in the following media with 10% (v/v) FBS (Bovogen Biologicals) and 1% (v/v) penicillin/streptomycin (10,000 U/mL, Gibco): HOSE 6‐3, HOSE 17‐1, HCT 116: DMEM/F12 (Gibco); SW620: DMEM/F12, 1% (v/v) HEPES (Gibco); all melanoma lines, MDA‐MB‐435, SW480: RPMI 1640(−Glutamine) (Gibco), 2 mM GlutaMAX (Gibco); MDA‐MB‐231: RPMI 1640(+Glutamine) (Gibco); OVCAR‐3, OVCAR‐5: DMEM high glucose (Gibco); A549, U‐87 MG, COLO 320: DMEM/F12 (Gibco), 2 mM GlutaMAX; DMS53: Waymouth (Gibco); PC‐3: RPMI 1640(−Glutamine) (Gibco); Fibroblasts: DMEM high glucose (Gibco), 50 μM 2‐mercaptoethanol, 250 μM L‐Asparagine (Gibco), 1 mM HEPES (Gibco).

### 2.2 Generation of drug‐resistant cell lines

The 5FU‐resistant SW620 colon cancer cells were generated by continuous culture in the presence of increasing concentrations of 5FU. Exponentially growing SW620 cells were initially treated with 1 μM 5FU and when they reached confluence passaged 1:10 into a fresh flask with increasing concentrations of 5FU on each passage as follows: 4, 6, 8, 10, 12, 15, 30, 50, 100 μM. The same process was followed for the Vorinostat‐resistant cells with cells initially treated with 1 μM Vorinostat which was increased on each passage with the following doses: 4, 6, 8, 10, 12, 15 μM.

### 2.3 CellTiter‐Glo viability assay

CellTiter‐Glo‐based viability assays were performed as previously described.^14^ Briefly, cells (2000 per well for 24 and 48 h timepoints, 500 cells per well for 120 h timepoint) were seeded into 96‐well white plates and 2–4 h later treated with serial dilutions of monepantel or equivalent vehicle (100% ethanol) to the highest concentration prepared in media used for each cell line. Cell viability was assessed after 24, 48, and 120 h treatment using the CellTiter‐Glo 2.0 assay (Promega, Australia) following the manufacturer's instructions. Luminescence was measured on an Ensight Multimode plate reader (Perkin Elmer). The results were normalized to the viability of cells treated with the highest % of vehicle. GraphPad Software was used to prepare graphs.

### 2.4 Flow cytometry apoptosis assay

Cells (6000–30,000 per well) were seeded into 24‐well plates, then 16–24 h later treated with monepantel or vehicle prepared in media used for each cell line. Live and dead cells were harvested and pelleted by centrifugation then incubated with Annexin V‐APC (BD Biosciences) and propidium iodide (PI) (Sigma Aldrich) in Annexin V binding buffer (BD Biosciences). FACS analysis was performed on a BD FACSCanto II flow cytometer (BD Biosciences). Data were analyzed using FlowJo Software Version 10 (FlowJo‐LLC) with the number of viable cells (Annexin V negative/PI negative) normalized relative to the number of viable cells cultured in the vehicle control.

### 2.5 3D cell culture

Cells (1 × 10^5^; LM‐MEL‐28, A549) from cultured cell lines were harvested, pelleted, and resuspended in 100 μL of Cultrex RGF BME, Type 2 (R&D Systems). Drops (4–8) of the suspension were then added to each well of a pre‐warmed 12‐well plate, then incubated inverted at 37°C for 30 min before media was added. Cells were then incubated at 37°C in 10% CO_2_ for 2–4 weeks. Media was then removed before cold TryPLE was added and cells resuspended by pipetting, then incubated for 10 min at 37°C before further shearing by pipetting. This resuspension process was then repeated before the wells are examined under a microscope to ensure a single‐cell suspension is obtained. Cells were then centrifuged at 320 *g* for 5 min, resuspended in Cultrex and split between two wells of a 12‐well plate with 3–4 domes per well. Spheroids were allowed to grow for ~1 week before media was changed into media containing the vehicle or 25 μM monepantel. Spheroids were imaged at Day 0 and 168 h posttreatment before FACS analysis.

### 2.6 3D flow cytometry assay

Media was removed from wells from 3D cultures and domes were individually pipetted and transferred to 300 μL of cold TryPLE and resuspended as described above then incubated with Annexin V‐APC (BD Biosciences) and propidium iodide (PI) (Sigma Aldrich) in Annexin V binding buffer (BD Biosciences). FACS analysis was performed on a BD FACSCanto II flow cytometer (BD Biosciences) and data were analyzed as described above.

### 2.7 Cell cycle analysis

Cell cycle analysis was performed similarly to the apoptosis assays. After drug treatment, cells were harvested, pelleted, and resuspended in PI buffer (50 μg/mL PI, 3.9 mM sodium citrate, 0.001% (v/v) Triton x‐100) prior to FACS analysis on a BD FACSCanto II flow cytometer using appropriate gating parameters for cell cycle. Data were analyzed using FlowJo Software Version 10 cell cycle platform (FlowJo‐LLC).

### 2.8 Western blotting

Total protein extracts were prepared by lysing cells (typically ~1–10 × 10^6^) for 1 h at 4°C in Onyx lysis buffer (20 mM Tris pH 7.4, 135 mM NaCl, 1.5 mM MgCl_2_, 1 mM EGTA, 10% (v/v) glycerol and 1% (v/v) Triton‐x‐100; Sigma‐Aldrich) for all blots except those probed with antibodies to CHOP and BiP where enhanced RIPA (50 mM Tris HCl pH 7.6, 150 mM NaCl, 1% (v/v) Igepal, 0.3% (w/v) sodium deoxycholate, 1% (w/v) SDS) or for 4EBP1 where Igepal buffer (50 mM Tris HCl pH 8.0, 150 mM NaCl, 1 mM EDTA, 1% (v/v) Igepal) was used instead. Lysis buffers were supplemented with protease inhibitors (Complete Mini, EDTA‐free (Roche)). Lysate supernatants (typically 50 μg total protein or equivalent to ~200,000 cells) were separated by SDS‐PAGE (NuPAGE 4%–12% Bis Tris gels, Invitrogen) before transferring to nitrocellulose and probing with antibodies to: BAK (Sigma, Cat# B5897‐2ML), BAX (WEHI, Clone3C10), ATG7 (Sigma, Cat#A2865), BiP (Cell Signaling Technologies, Cat#3183S), CHOP (Cell Signaling Technologies, Cat#2895S), actin (Sigma, Cat#A2228‐200UL), p62 (Cell Signaling Technologies, Cat#5114S), LC3 (Novus, Cat#NB100‐2220), 4EBP1 (Cell Signaling Technologies, Cat#9644), NOXA (Abcam, Cat#ab13654).

### 2.9 RNA sequencing

Cell lines (LM‐MEL‐28, A549, HOSE 6‐3 or OVCAR‐3) were seeded into 6‐well plates (1 × 10^5^ cells/well) and allowed to adhere overnight. The media was then replaced with media containing 25 μM monepantel or an equivalent volume of vehicle (ethanol) and incubated at 37°C for 48 h. Cells were collected and counted and pellets containing 1 × 10^5^ cells snap‐frozen prior to total RNA extraction performed using the miRNeasy micro kit (Qiagen) following the manufacturer's instructions. The RNA concentration was determined using RNA ScreenTape on the TapeStation instrument (Agilent).

RNA‐Seq libraries were prepared using the Illumina TruSeq RNA protocol on 80 ng of purified RNA. Briefly, mRNA was isolated and purified through binding of poly‐A RNA to oligo dT magnetic beads. The first strand and second strand cDNA were synthesized from fragmented mRNA then the cDNA underwent end repair and A‐tailing before RNA adapter ligation. The indexed adaptors were enriched by PCR for 15 cycles. The quantity of DNA was determined using D5000 tape on the Tapestation instrument (Agilent). RNAseq libraries were pooled prior to Next Gen Sequencing on an Illumina NextSeq500 platform performed at the Walter and Eliza Hall Institute (Melbourne, Australia).

### 2.10 RNA‐seq data analysis

Sequencing reads were mapped to the GRCh38/hg38 reference genome using the *Subread* aligner in the *Rsubread* package.^15^, ^16^ Only uniquely mapped reads were retained. Gene‐wise read counts were obtained using the *featureCounts* program.^17^ Only genes that achieved a counts per million (CPM) value greater than 1 in at least two samples were included in the downstream analysis. Read counts were converted to log2‐CPM, quantile‐normalized, and precision‐weighted with *voom* function in *limma* package.^18^, ^19^ Log2‐CPM values were then converted to log2‐FPKM (Fragments Per Kilobases per Million at log2 scale) values for each gene. A linear model was fitted to each gene and empirical Bayes moderated *t*‐statistic was used to assess differential expression of genes.^20^ Genes were called differentially expressed (DE) if they achieved a false discovery rate (FDR) of 0.05 or less. The *goana* and *kegga* functions^21^ in *limma* were used to test the enrichment of GO (Gene Ontology) terms and KEGG (Kyoto Encyclopedia of Genes and Genomes) pathways, respectively, in cell lines treated with monepantel compared to vehicle controls. The *barcodeplot* function in *limma* was used to generate barcode plots displaying enrichment of KEGG pathways of particular interest. Statistical significance of enrichment of these pathways was tested using the *limma* function *roast*.^22^

### 2.11 CRISPR deletion of ATG7

Deletion of ATG7 was performed using a lentiviral vector system^23^ employed as described previously.^14^ sgRNAs were designed using CRISPick design software (portals.broadinstitute.org/goox/crispick/public) providing the sequences: (1) 5′‐GAAGCTGAACGAGTATCGGC‐3′ and (2) 5′‐GCTGCCAGCTCGCTTAACAT‐3′. Expression of sgRNA was induced by treatment with doxycycline (1 μg/mL, Sigma) for at least 3 days prior to Western blotting to determine gene deletion efficiency.

### 2.12 Statistical analysis

For all apoptosis assays where treatments resulted in >10% change in cell viability, statistical analysis was performed to compare vehicle versus treatments or corresponding treatments between cell genotypes (i.e., with WT versus *Bak* ^−/−^ *Bak* ^−/−^ or *Atg7* ^−/−^ cells) with Prism 8 (GraphPad) software using an unpaired t‐test with Welch's correction. In the case of titrations, the same test was applied to data corresponding to any individual doses where there was an obvious difference in response between cell lines, or using Wilcoxon matched‐paired signed rank test to compare entire dose–response curves.

## 3 RESULTS

### 3.1 Monepantel has activity across a wide range of cancer cell lines and types

Other than a recent study where we tested monepantel on a small number of cell lines,^13^ essentially all other studies on the anti‐cancer activity of monepantel have been performed on ovarian cancer. To gain some further insight into its pan‐cancer activity, we examined monepantel in CellTiter‐Glo (CTG) luminescent viability assays in over 20 cell lines ranging across melanoma, lung, breast, brain, colorectal, prostate, and ovarian, as well as SV40 Large T‐transformed mouse embryonic fibroblasts (MEFs). Non‐malignant, human ovarian surface epithelial cells (HOSE 6‐3 and HOSE 17‐1) were also tested as previous studies had suggested these were less sensitive compared to cancer lines.^12^

**FIGURE 1:**
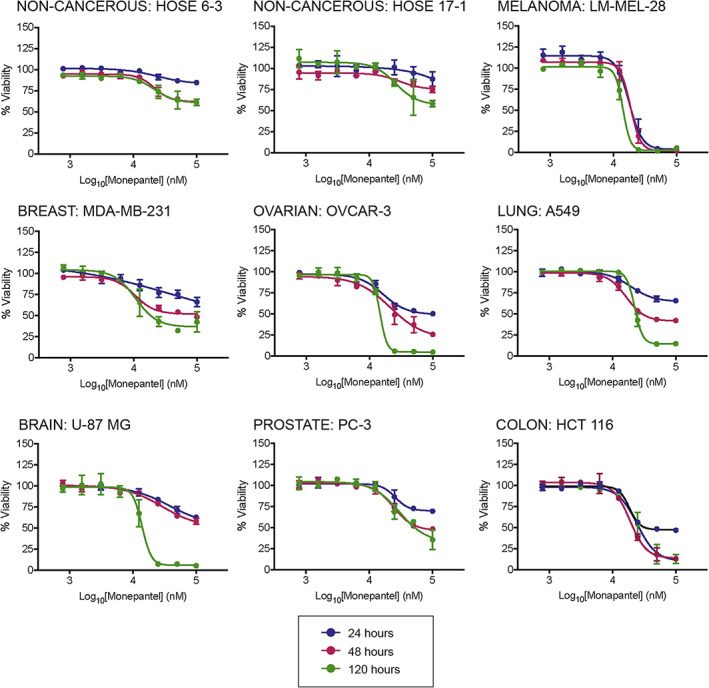
Monepantel treatment reduces cell viability across a range of cancer types. Representative cell lines were treated with monepantel for 24–120 h then viability measured using CTG assays. Values shown are mean ± SEM of *n* = 3 experiments. Data for all cell lines tested are available in Figures S1 and S2.

Overall, a relatively consistent dose‐dependent reduction in cell viability was observed across all lines, with the effect increasing with time (Figure 1, Figures S1 and S2). The EC_50_ values at the 48 and 120 h timepoints ranged from 10 to 25 μM, consistent with previous studies.^9^, ^11^, ^12^ No single cancer type was found to be overtly sensitive to the drug, while both non‐malignant lines were among the least sensitive, as previously observed.^12^ To test if resistance to other cancer drugs inhibits monepantel efficacy, we first continuously passaged SW620 colon cancer in drugs used for colon cancer treatment, Vorinostat and 5FU, so that they became 3‐ to 30‐fold resistant to each drug, respectively (Figure S2A,B). Upon treatment with monepantel, resistance to 5‐FU resulted in significantly decreased efficacy, but only at the shorter time points (24 and 48 h). Notably, no significant effect was seen with 120 h treatment. In the Vorinostat‐resistant cells, no significant decrease in monepantel efficacy was observed.

In summary, these data demonstrate monepantel has broad activity on solid cancer cell lines and could potentially be advantageous in cases where resistance to other standard treatments occurs.

### 3.2 Apoptosis pathways are not required for monepantel activity

Previous studies in ovarian cancer cell lines have shown that monepantel fails to induce extensive apoptosis but instead induces autophagic cell death.^12^ However, its effects on apoptosis in other solid cancer types is unknown.

**FIGURE 2:**
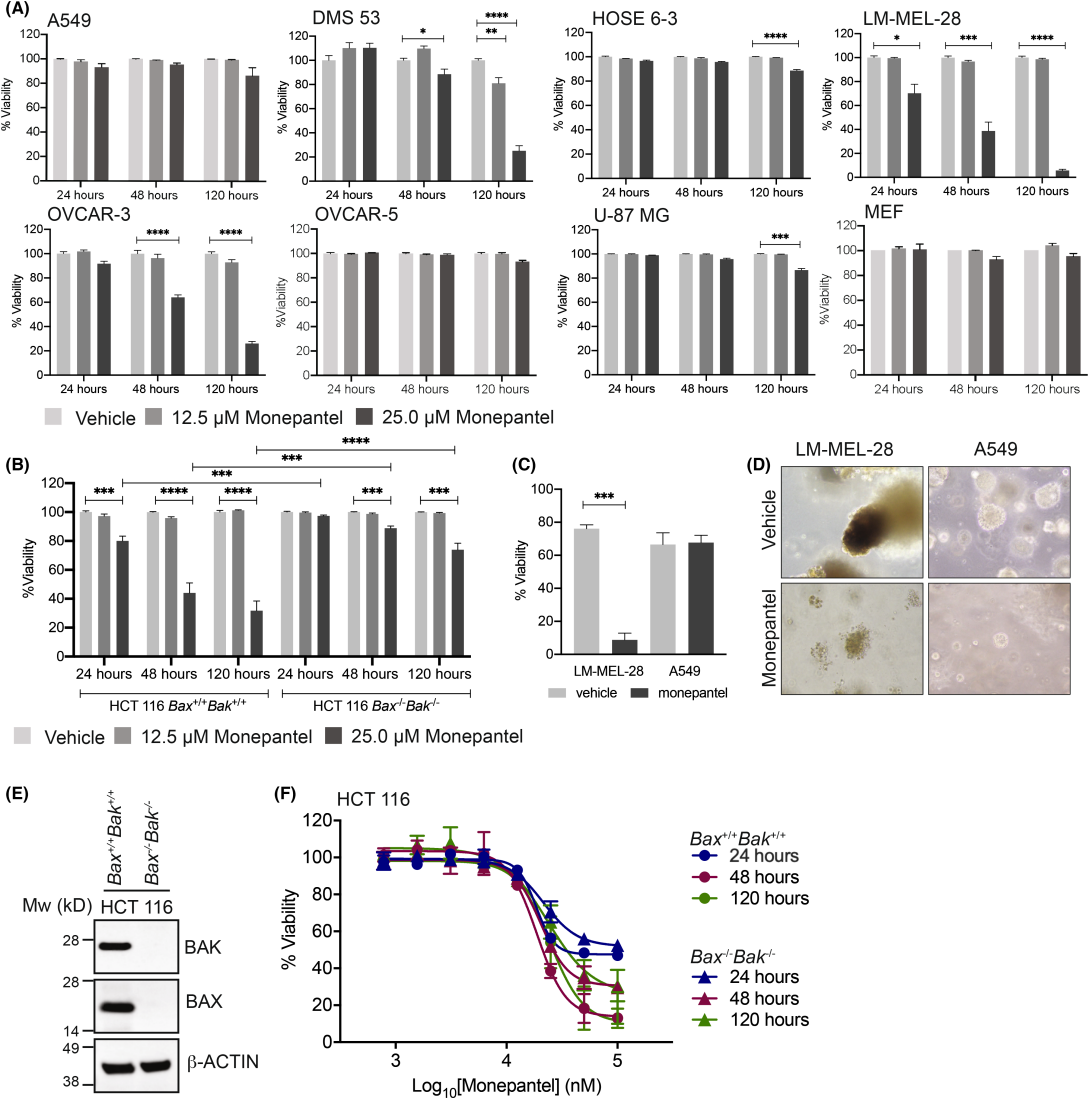
Monepantel induces apoptosis in some but not all cell lines. (A) Representative cell lines were treated for 24–120 h with the indicated doses of monepantel and then analyzed for apoptosis induction by FACS following staining with Annexin V and PI. Values shown are mean ± SEM of *n* = 3 experiments. (B) HCT 116 cells show evidence of apoptosis, but this is significantly attenuated in the absence of BAX and BAK. Values shown are mean ± SEM of *n* = 4 experiments. (C) Spheroid cultures of LM‐MEL‐28, but not A549 cells undergo apoptosis following treatment with 25 μM monepantel for 168 h (***p = 0.006). (D) Representative images of the spheroids after treatment with vehicle or monepantel. (E) Western blot confirming deletion of BAX and BAK in *BAX* ^−/‐^ *BAK* ^−/−^ HCT 116 colorectal cancer cells. β‐Actin was used as a loading control. (F) Deletion of BAX and BAK has only a minor impact on cell viability in CTG assays following monepantel treatment in HCT 116 cells. Values shown are mean ± SEM of *n* = 3 experiments. *p = 0.01–0.05, **p = 0.001–0.01, ***p = 0.0001–0.001, ****p < 0.0001.

To address this, apoptosis induction was assessed in a representative panel of cancer and non‐malignant cell lines. Strong Annexin V staining was observed in some (LM‐MEL‐28 (melanoma), DMS 53 (lung), OVCAR‐3 (ovarian), HCT 116 (colon)) but not other lines (OVCAR‐5 (ovarian), A549 (lung), U‐87 MG (brain), MEF (mouse embryonic fibroblast)), including the HOSE 6‐3 line that showed only a weak response in the CTG assay (Figure 2A,B). In all cases where Annexin V staining was observed (LM‐MEL‐28, OVCAR‐3, DMS 53, HCT 116) there was evidence of early or intermediate apoptotic subpopulations where cells did not stain maximally for PI (Figures S3A–I and S4A). This is unlike previous reports that only detected late apoptotic/necrotic cells following monepantel treatment.^12^

We also extended our studies to more physiologically relevant 3D cultures. Here, we generated spheroids of LM‐MEL‐28 cells that undergo significant apoptosis in 2D culture, as well as A549 cells that only showed decreased proliferation with minimal apoptosis. Both 3D cultures responded to monepantel very similarly to how they responded in the 2D cultures; the LM‐MEL‐28 cells underwent apoptosis, while the A549 monepantel‐treated spheroids remained as viable as those treated with vehicle (Figures 2C,D; Figure S4B) but grew more slowly and were not as large as those treated with the vehicle at the end of the experiment suggesting proliferation was inhibited. Notably, the LM‐MEL‐28 cells grew as pigmented melanoma spheroids, and this pigmentation was lost following monepantel treatment (Figure S4C).

As HCT 116 colon cancer cells showed obvious early/intermediate apoptotic populations following monepantel treatment suggestive of apoptosis induction rather than necrosis or some other mode of cell death, we next tested the effect of monepantel on an isogenic derivative of HCT 116 cells that were doubly deficient (“DKO”) for BAX and BAK (*BAX* ^*−/−*^ *BAK* ^*−/−*^ cells) (Figure 2B,E). BAX and BAK are the essential “executors” of the intrinsic apoptosis pathway,^24^ hence, apoptosis is significantly reduced in their absence, thereby enabling confirmation of an apoptotic response to treatment. Indeed, the apoptotic populations significantly decreased in the DKO cells at all timepoints with 25 μM treatment (Figure 2B; Figure S4A). Consistent with these findings, the viability of the DKO cells was marginally increased in the CTG assays relative to the wild‐type cells after 48‐ and 120‐h treatment with high monepantel doses, though this was not significant (Figure 2F). This suggests that while monepantel induces apoptosis in the absence of BAX and BAK, cells still fail to proliferate even when apoptosis is suppressed.

### 3.3 Autophagy is not required for monepantel activity

**FIGURE 3:**
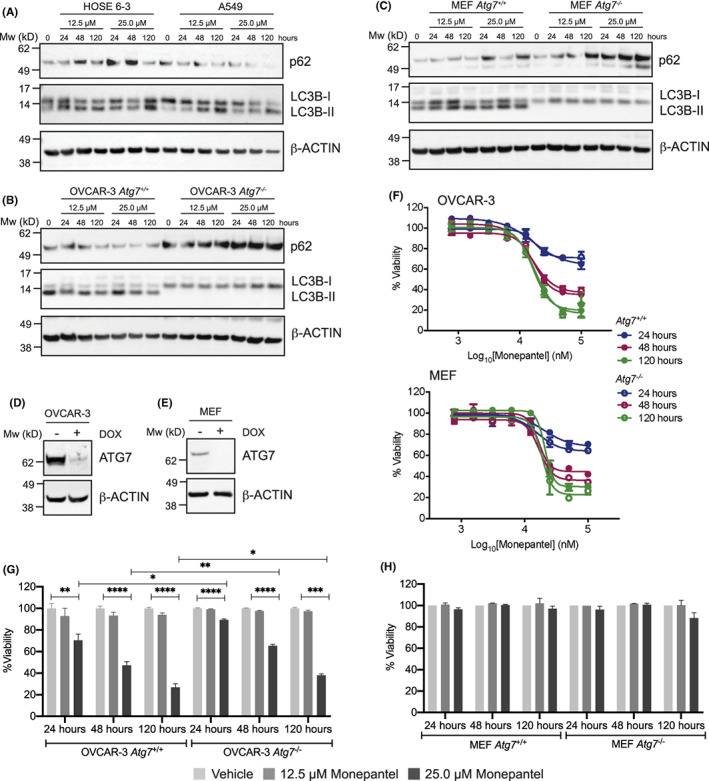
Autophagy is not required for the anti‐proliferative activity of monepantel. (A–C) Western blots for autophagy markers of indicated cell lines following monepantel treatment at indicated timepoints and doses. Quantitation of LC3B blots is shown in Figure S5. (D, E) Western blots showing deletion of ATG7 following doxycycline (DOX) treatment in indicated cell lines. β‐actin was used as a loading control in all Western blots. (F) CTG viability assays on the indicated cell lines that were either wild‐type (*ATG7* ^*+/+*^) or KO (*ATG7* ^*−/−*^). Values shown are mean ± SEM of *n* = 3 or 4 experiments. G, H) FACS‐based apoptosis assays on the indicated cell lines that were either ATG7 wild‐type or KO. Values shown are mean ± SEM of *n* = 3 experiments. *p = 0.01–0.05, **p = 0.001–0.01, ***p = 0.0001–0.001, ****p < 0.0001.

As previous studies had proposed that autophagic cell death was involved in the anti‐tumor activity of monepantel in ovarian cancer cell lines,^12^ we next examined whether similar effects were induced in other tumor types. As expected, monepantel induced autophagy in HOSE 6‐3 and A549 cells as well as MEFs, evidenced by a change in the ratio of LC3B‐I to LC3B‐II relative to the 0 h time point (Figure 3A,C; Figures S5A,B,E). In OVCAR‐3 cells which had high basal levels of LC3B‐II relative to LC3B‐I, this ratio did not change much though absolute levels of LC3B‐II were reduced (Figure 3B, Figure S5C). To determine whether autophagy was *required* for monepantel activity, we used CRISPR‐Cas9 gene editing to generate OVCAR‐3 and MEF cells that were deficient in ATG7 (*ATG7* ^*−/−*^), an enzyme required for lipidation of LC3B‐I, converting it to LC3B‐II, an essential step in autophagosome maturation. Both *ATG7* ^*−/−*^ lines showed significantly decreased ATG7 protein levels (Figures 3D,E) compared to their wild‐type counterparts, and a complete absence of LC3B‐II consistent with efficient and functional deletion of ATG7 (Figure 3B,C; Figure S5D,F). Notably, both *ATG7* ^*−/−*^ cell lines behaved identically to the parental lines in CTG assays in response to monepantel treatment (Figure 3F). Similar results were observed in the FACS‐based apoptosis assays, though the OVCAR‐3 *ATG7* ^*−/−*^ cells were marginally less sensitive to monepantel compared to their wild‐type counterparts at each time point (Figure 3G; Figure S6A). Although MEFs failed to undergo apoptosis in response to monepantel (Figure 2A), deletion of ATG7 did not make them more sensitive to the drug (Figure 3H; Figure S6B). Both ATG7^*−/−*^ lines showed obvious accumulation of p62 in response to monepantel, which was not observed in the wild‐type cells (Figures 3B,C), further demonstrating that monepantel impacts the autophagy pathway. Collectively, these data strongly suggest that while autophagy is induced in response to monepantel treatment, it is not required for its anti‐proliferative activity.

### 3.4 Transcriptomic analysis reveals key pathways induced and repressed by monepantel treatment

To provide an unbiased analysis of the mechanism‐of‐action of monepantel, bulk RNA‐sequencing was performed on four cell lines that demonstrated different responses across CellTiter‐Glo and FACS assays: HOSE 6‐3 cells which were relatively resistant to monepantel treatment, A549 cells that responded well in the CTG assay but not the FACS assay, and LM‐MEL‐28 and OVCAR‐3 cells which showed strong responses in both assays. In all cases, cells were treated with 25 μM monepantel for 48 h (a timepoint where strong responses were typically initially observed in CTG assays), prior to RNA isolation and sequencing.

**FIGURE 4:**
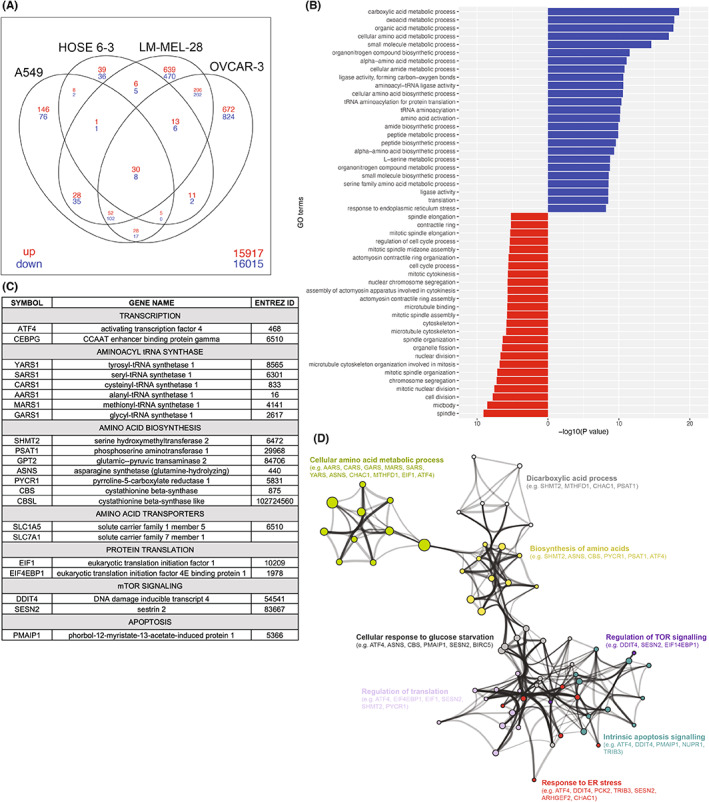
Transcriptomics analysis of four cell lines following monepantel treatment. (A) Venn diagram showing unique and overlapping differentially expressed genes (monepantel treated versus vehicle control) for all four cell lines. (B) Barplots for the top 25 GO terms enriched in the differentially expressed genes up‐regulated (blue bars) and down‐regulated (red bars) in all four cell lines. (C) Genes related to ER‐stress including those involved in amino acid synthesis and metabolism, apoptosis and mTOR signaling upregulated in all four cell lines. (D) Network map showing connections between major GO and KEGG terms for upregulated differentially expressed genes in all four cell lines.

Multi‐dimensional scaling of the data revealed clustering of treated and untreated samples from the same cell lines (Figure S7A). Differentially expressed gene (DEG) analysis revealed that the number of genes upregulated versus those downregulated was approximately the same within each cell line, although the total number of DEGs varied greatly between cell lines (Figure 4A). Interestingly, there was a positive correlation between drug responses and the extent of transcriptional changes with fewer DEGs in the weaker‐responding lines compared to the cell lines where greater responses were observed: HOSE 6‐1: 60/113 down/up, A549: 241/298 down/up, LM‐MEL‐28: 829/975 down/up, OVCAR‐3: 1161/1017 down/up (Figure 4A). While there was a large number (>600) of genes uniquely differentially expressed in each of the highly responsive lines (OVCAR‐3 and LM‐MEL‐28), many DEGs were also shared between the lines. For example, 206/202 genes were similarly up/downregulated in OVCAR‐3 and LM‐MEL‐28 cells and 52/102 genes were similarly up/down‐regulated in the three most responsive lines, indicating overlapping effects across cancer types.

We further interrogated the 30 commonly upregulated and 8 commonly downregulated genes across all four cell lines by gene ontology (GO) analysis (Table S1). Of the commonly upregulated genes, GO analysis revealed enrichment of processes related to amino acid metabolism including various enzymes associated with amino acid synthesis or modification as well as amino acid transporters and sensors (Figure 4B, Table S2). Specific upregulated genes included *ATF4*, a master transcription factor associated with ER stress responses, *EIF1*, *EIF4EPB1* and six different amino acid tRNA synthetase 1 genes which are associated with regulation of protein synthesis, and *DDIT4* and *SESN2* which are associated with mTOR signaling (Figure 4C). Combined, these data suggest a significant impact on multiple interconnected signaling pathways across all cell lines following monepantel treatment (Figure 4D).

The eight commonly downregulated genes were predominantly associated with cell cycle regulation consistent with our data that monepantel impacts cell proliferation. Moreover, barcode plots generated for individual cell lines following enrichment analysis showed strong negative enrichment for genes associated with cell cycle following monepantel treatment (Figure S7B).

**FIGURE 5:**
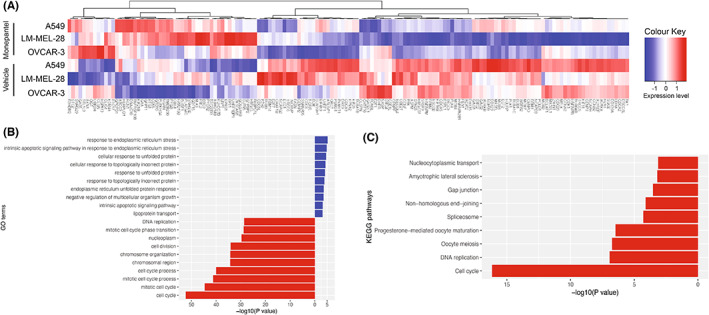
Gene expression analysis of the three cell lines (A549, LM‐MEL‐28, OVCAR‐3) sensitive to monepantel treatment. (A) Heatmap showing differentially expressed genes in the sensitive cell lines but *not* HOSE 6‐3. Relative expression levels (Z‐scores) of genes are shown in the heat maps, color‐coded according to the legend. Rows are scaled to have a mean of 0 and an s.d. of 1. (B) Barplots for the top 10 GO terms enriched in the differentially expressed genes up‐regulated (blue bars) and downregulated (red bars) in the three sensitive cell lines but not in the HOSE 6‐3 cell line. (C) Barplots for the top KEGG terms enriched in the differentially expressed genes downregulated (red bars) in the three sensitive cell lines but not in cell line HOSE 6‐3.

To gain potential insights into the molecular basis for the differential sensitivity of cell lines to monepantel, we next identified genes differentially regulated in the most responsive cell lines (A549, LM‐MEL‐28 and OVCAR‐3) but not in the HOSE 6‐3 cell line (Figure 5A). This significantly increased the number of genes in our analysis, especially for down‐regulated genes (52 upregulated and 102 downregulated) (Figure 3A, Table S3). The gene ontology (GO) analysis of the upregulated genes again identified the ER stress/unfolded protein response as being induced in response to monepantel treatment (Figure 5B, Table S4), while GO and KEGG pathway analysis identified multiple terms associated with cell cycle regulation (Figure 5B,C, Table S4).

Hence, the RNAseq data strongly points to two major cellular processes with monepantel treatment—the ER stress pathway and cell cycle regulation.

### 3.5 Validation of the involvement of cell cycle, apoptosis and ER stress proteins in the mechanism‐of‐action of monepantel

**FIGURE 6:**
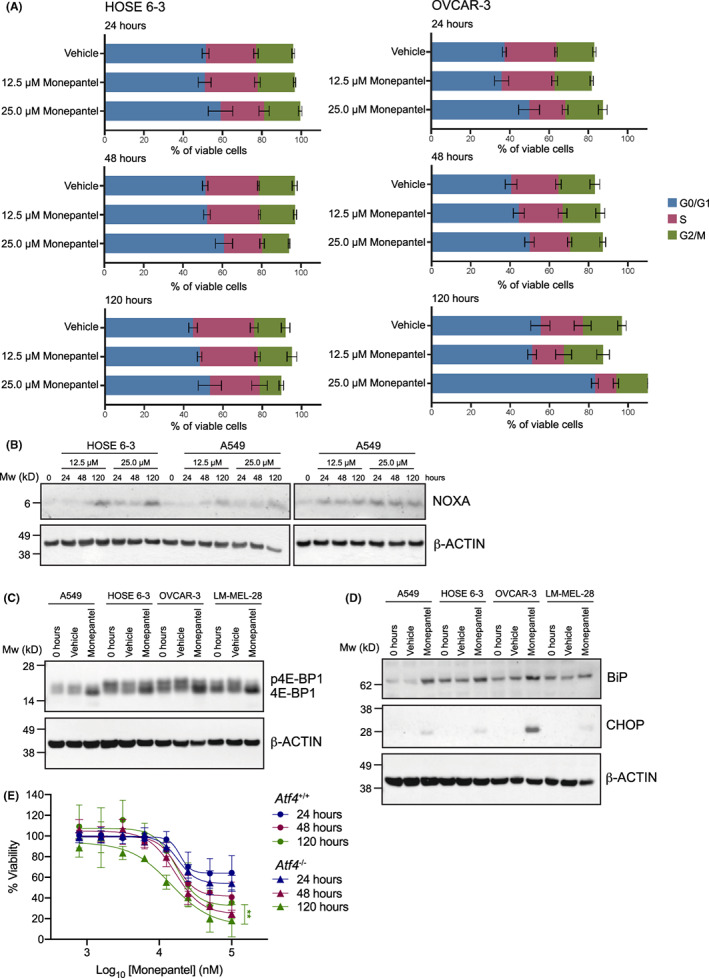
Validation of transcriptomics data. (A) Cell cycle analysis shows an increase in the G0/G1 population following monepantel treatment in OVCAR‐3 cells. Values shown are mean ± SEM of *n* = 2 experiments. (B) Western blot analysis shows an increase in NOXA levels in the four indicated cell lines, consistent with the transcriptomics data. (C) Western blot analysis shows an increase in 4E‐BP1 (hypophosphorylated form) in the four indicated cell lines, consistent with the transcriptomics data. (D) Western blots showing BiP and CHOP proteins are upregulated following monepantel treatment, consistent with cells undergoing ER stress. (E) MEFs deficient for Atf4 (*Atf4* ^*−/−*^) are more sensitive to monepantel treatment compared to their wild‐type (*Atf4* ^*+/+*^) counterparts. Cell lines were treated with monepantel for 24–120 h then viability measured using CTG assays. Values shown are mean ± SEM of *n* = 3 experiments (**p = 0.001–0.01).

To functionally validate the downregulation of cell cycle genes, particularly in the most responsive cell lines, we next examined the effect of monepantel on cell cycle kinetics. Consistent with the gene expression changes, monepantel induced an increase in the percentage of cells in G0/G1 and a reduction in the percentage of cells in S phase in sensitive OVCAR‐3 cells. While a similar trend was observed in HOSE 6‐3 cells, effects were less pronounced (Figure 6A). As this effect of monepantel treatment on expression of cell‐cycle regulators has been previously examined,^10^, ^11^ we instead focused on the upregulated genes and pathways as these provided a potentially novel insights into mechanism‐of‐action of monepantel.

We next examined induction of *PMAIP* which encodes the pro‐apoptotic BH3‐only protein NOXA, which was upregulated by monepantel in all cell lines irrespective of apoptotic response (Table S1). Consistent with the transcriptomic data, Western blotting confirmed increased NOXA expression in all cell lines tested at most time points and at both concentrations (12.5 and 25 μM) (Figure 6B). It should be noted that NOXA has a very specific BCL‐2 protein binding profile, only targeting MCL‐1 and BFL‐1 but not BCL‐2, BCL‐XL, or BCL‐W. Hence, whether the cells undergo apoptosis in response to NOXA induction will depend on the relative levels of these and other BCL‐2 family members which could explain the differential responses among the cell lines.

**FIGURE 7:**
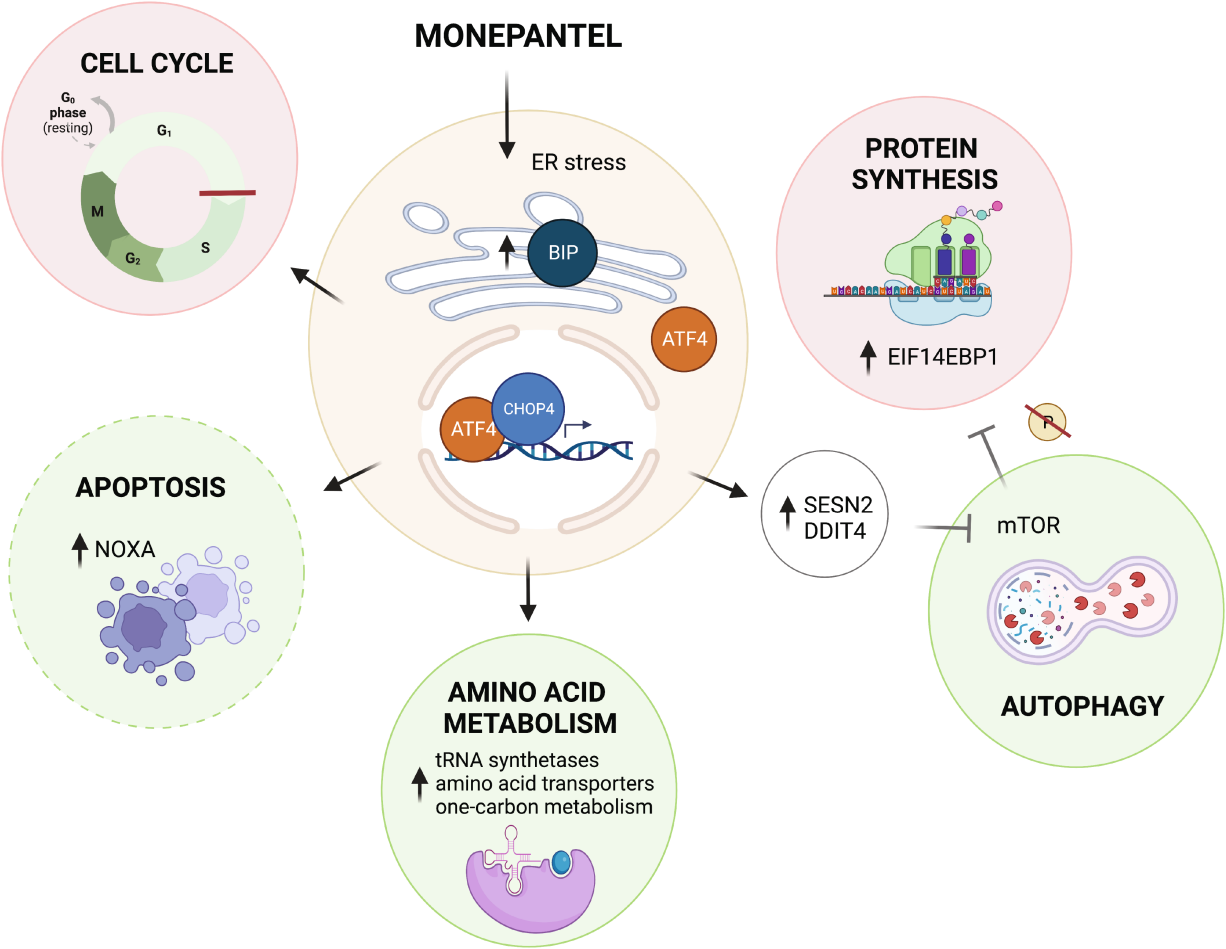
Schematic showing how monepantel activates multiple pathways to elicit its anti‐cancer effects. Processes in red circles are downregulated while those in green circles are upregulated.

The GO analysis strongly implicated ER stress pathways being upregulated in response to monepantel treatment. One consequence of ER stress is the suppression of global translation which is regulated by 4E‐BPs that inhibit the assembly of the eIF4F complex essential for translation initiation in eukaryotes. The 4E‐BPs are transcriptionally upregulated by ATF4,^25^ which was observed in the RNAseq analysis (Table S1) and confirmed at the protein level by Western blotting at all timepoints (24–120 h) (Figure 6C). Notably, the hypophosphoryated form (i.e., faster migrating) of 4E‐BP1 which binds to the eIF4E complex with high affinity (unlike the phosphorylated form) is the predominant species observed. Other well‐known ER stress markers such as BiP and CHOP were also upregulated following monepantel treatment in all cell lines (Figure 6D). To test the importance of ATF4 induction in eliciting the growth inhibitory effect of monepantel, we treated MEFs in which *Atf4* was deleted (*Atf4* ^*−/−*^) with monepantel (Figure 6E). While *Atf4* deletion had minimal impact on cell viability at 24–48 h, sensitivity to monepantel was significantly increased at 120 h, suggesting that ATF4‐mediated upregulation of ER stress pathways may be a survival mechanism in response to monepantel (Figure 7).

## 4 DISCUSSION

Previous studies on the mechanism‐of‐action of monepantel have predominantly pointed to autophagy, mTOR signaling and the cell cycle as critical pathways associated with the anti‐cancer effects of the drug, though apoptosis has also been implicated.^9^, ^10^, ^11^, ^12^ However, these studies have exclusively been undertaken in ovarian cancer cell lines. In this current report, we have used a candidate‐based genetic approach targeting key pathways combined with transcriptomic analysis to provide new insights into how monepantel acts to suppress cancer cell growth in a range of cancer types.

We first showed that monepantel has broad anti‐cancer effects across multiple cancer types (melanoma, lung, breast, brain, colorectal, prostate, and ovarian) with similar dose (IC_50_s in the 10–25 μM range) and time‐dependent (48–120 h) effects on cell viability across the cell lines tested. As previously reported, non‐malignant cell lines tested were generally less sensitive compared to the majority of cancer lines examined. Interestingly, we observed evidence of apoptosis induction in approximately 50% of a representative subset of cell lines examined which is inconsistent with previous studies that concluded apoptosis is not induced by monepantel. The use of BAX/BAK‐deficient cells has become the gold standard for confirming whether drugs are acting through the BCL‐2‐regulated pathway.^26^ Our data implicating apoptotic cell death as a consequence of monepantel treatment is supported by evidence from these knockout cells where the apoptotic response was largely ablated. However, while the apoptotic response of these cells was suppressed, cell proliferation was only marginally affected. This suggests that while apoptosis induction is a potential consequence of monepantel treatment, it is not the primary mechanism by which monepantel exerts its anti‐cancer effect and that it more likely involves inhibition of the cell cycle. Transcriptional analysis provided further evidence for the effect of monepantel on cytokinesis. Indeed, in those cell lines analyzed that were most responsive to the drug, the vast majority of the dozens of genes downregulated were associated with cell cycle regulation, and consistent with previous studies, we showed that monepantel treatment arrested cells in G0/G1.

The RNAseq data, however, also provided compelling new evidence for the involvement of an ER stress response in the activity of monepantel including the upregulation of ATF4, a master regulator of ER stress responses, and multiple genes associated with amino acid (especially serine) metabolism. At a protein level, we observed further evidence of an ER‐stress response including increased levels of the ER chaperone protein BiP and the major transcription factor acting downstream of ATF4, CHOP.^27^, ^28^ Furthermore, the gene transcription pattern strikingly matches transcriptional signatures and the GO terms enriched following treatment with potent ER‐stress inducers such as thapsigargin and tunicamycin in multiple other studies.^29^, ^30^, ^31^ In addition to ATF4, some of the specific genes upregulated include a large number of amino acid tRNA synthetases/amino acid synthetase (AARS, CARS, GARS, MARS, SARS, YARS, ASNS), one‐carbon metabolism genes (SHMT2, MTHFD2), and amino acid transporters (SLC1A5, SLC7A1) which are all well‐characterized genes associated with ER stress.^29^, ^30^, ^31^, ^32^

Notably, deletion of ATF4 increased the sensitivity of MEFs to monepantel, strongly suggestive of ER stress being induced as a survival mechanism, presumably elicited by the unfolded protein response (UPR) associated with ER stress.^33^ Hence, while the transcriptional signature we observe following monepantel treatment implicates the ATF4 ER stress pathway, other stress pathways are likely upregulated to compensate for the absence of ATF4. Notably, both the ATF6‐ and IRE1a‐mediated ER stress pathways that also act downstream of BiP can also significantly impact cell proliferation.^34^, ^35^ Prolonged ER stress can also lead to cell death, including through the upregulation of NOXA which we observed transcriptionally and at the protein level, though this can occur in an ATF4‐independent manner.^29^, ^36^, ^37^, ^38^, ^39^

One consequence of the UPR is the global suppression of protein translation which is mediated by the 4E‐BPs (direct targets of ATF4) that inhibit eIF4F assembly required for translation initiation.^25^ Notably, the *EIF4EBP* gene was transcriptionally upregulated, as was the encoded 4E‐BP1 protein, in response to monepantel treatment. Other reports on the anti‐cancer effects of monepantel place 4E‐BP1 along the mTOR signaling axis and induction of autophagy as critical processes underlying drug activity. Consistent with this, our data show upregulation of two critical negative regulators of mTORC1, *DDIT4*,^40^, ^41^ and *SESN2* ^42^ (also target genes of ATF4) in all cell lines. mTORC1 tightly regulates autophagy by suppressing its induction via phosphorylation‐dependent inhibition of ULK1/2. Hence, down‐regulation of mTORC1 activity results in autophagy, as we (and others) observed. Decreased mTORC1 activity results in reduced phosphorylation of 4E‐BP1,^43^ which we also observe, together with its transcriptional upregulation resulting in a significant shift in the hypophosphorylated:phosphophorylated 4E‐BP1 ratio, and consequently suppression of protein translation.

As in previous reports, we showed evidence of autophagy induction triggered in response to monepantel treatment. However, those previous studies did not provide any details on why or how it was induced. The compelling evidence reported here on the induction of ER stress responses likely fills that gap. Indeed, autophagy induction and mTOR signaling pathways are major consequences of ER stress.^44^, ^45^, ^46^, ^47^ Although previous reports have strongly implicated autophagic cell death as an outcome of monepantel treatment, our data using autophagy‐deficient ATG7 knock‐out cell lines suggests that this process probably has only a minor role in the anti‐cancer effect of monepantel as these lines behaved very similarly to their wild‐type counterparts. Nevertheless, it is clear that autophagy induction is associated with the activity of monepantel but this likely occurs downstream of ER stress, probably as a potential survival mechanism.

In summary, our data provide compelling evidence for the induction of ER stress and associated UPR as being involved in the mechanism‐of‐action of monepantel, and strongly suggests that cell cycle arrest is the major consequence of treatment, although apoptosis and autophagy can both occur downstream of the stress response (Figure 7). What is still missing is the knowledge of how monepantel triggers ER stress. Nevertheless, the data presented here will enable future studies directed toward the identification of the molecular target of monepantel in mammalian cells and a full understanding of its mechanism‐of‐action.

## AUTHOR CONTRIBUTIONS

**Tiffany J. Harris:** Data curation (equal); formal analysis (equal); investigation (lead); writing – review and editing (supporting). **Yang Liao:** Formal analysis (supporting); visualization (supporting); writing – original draft (supporting); writing – review and editing (supporting). **Wei Shi:** Formal analysis (supporting); visualization (supporting); writing – original draft (supporting); writing – review and editing (supporting). **Marco Evangelista:** Investigation (supporting); writing – review and editing (supporting). **Bhupinder Pal:** Formal analysis (supporting); writing – original draft (supporting); writing – review and editing (supporting). **Hamsa Puthalakath:** Resources (equal); writing – original draft (supporting); writing – review and editing (supporting). **Roger Aston:** Resources (supporting); writing – review and editing (supporting). **Richard Mollard:** Funding acquisition (supporting); resources (supporting); writing – original draft (supporting); writing – review and editing (supporting). **John M. Mariadason:** Resources (supporting); writing – original draft (supporting); writing – review and editing (supporting). **Erinna F. Lee:** Conceptualization (lead); data curation (lead); formal analysis (lead); funding acquisition (lead); methodology (lead); project administration (lead); resources (lead); supervision (lead); visualization (lead); writing – original draft (lead); writing – review and editing (lead). **Walter D. Fairlie:** Conceptualization (lead); data curation (lead); formal analysis (lead); funding acquisition (lead); investigation (equal); methodology (equal); project administration (lead); resources (lead); supervision (lead); visualization (lead); writing – original draft (lead); writing – review and editing (lead).

## Supporting information


Figure S1‐S7

Table S1

Table S2

Table S3

Table S4

## Data Availability

The data that support the findings of this study are available from the corresponding author upon reasonable request.
