# Supplementary material for: Induction of endoplasmic reticulum stress is associated with the anti‐tumor activity of monepantel across cancer types
Source: Cancer Med. 2023 May 6;12(12):13522–37. doi: 10.1002/cam4.6021 (PMC10315739; doi:10.1002/cam4.6021)
Supplement: Supplementary file 1 — Figure S1‐S7 [file CAM4-12-13522-s005.pdf]

Supplementary Materials

Supplementary Table 1: **Genes transcriptionally upregulated and downregulated in four cell lines following treatment with monepantel.**

Supplementary Table 2: **Common GO and KEGG terms enriched in four cell lines following treatment with monepantel.**

Supplementary Table 3: **Common genes upregulated in three cell lines sensitive to monepantel but not in HOSE 6-3.**

Supplementary Figure 1: **Monepantel treatment reduces cell viability across a range of cancer types.** The indicated cell lines were treated with monepantel for 24-120 hrs then viability measured using CTG assays. Values shown are mean±SEM of n=3 experiments.

Supplementary Figure 2: **Monepantel is effective on cell lines resistant to other cancer drugs.** SW620 colon cancer cells were made resistant to **A)** 5FU and **B)** Vorinostat. The efficacy of monepantel was only significantly affected at the shorter treatment times with **C)** the 5FU-resistant cells but not with **D)** the Vorinostat-resistant line.

Supplementary Figure 3: **Monepantel induces apoptosis in some cell lines. A)** Representative FACS plots for LM-MEL-28 cells treated with vehicle or 25 μM monepantel for 120 hrs showing how the different populations of live, apoptotic and necrotic cells were distinguished after staining for Annexin V and PI. **B-I)** Analysis of the proportion of live, apoptotic (early, intermediate, late) and necrotic cell populations for the indicated cell lines following 24, 48, 120 hrs treatment with monepantel (MPL) or vehicle. Data is the mean±SEM of n=3 experiments.

Supplementary Figure 4: **Apoptosis analysis in BAX/BAK-deficient cells and 3D cultures. A)** Analysis of the proportion of live, apoptotic (early, intermediate, late) and necrotic cell populations (as distinguished in Supplementary Figure 3) for wild-type and BAX/BAK-deficient HCT 116 cells following 24, 48, 120 hrs treatment with monepantel (MPL) or vehicle. Data is the mean±SEM of n=3 experiments. B) Analysis of the proportion of live, apoptotic (early, intermediate, late) and necrotic cell populations for the indicated cell lines grown as 3D

cultures following 120 hrs treatment with 25 μM monepantel or vehicle. Data is the mean±SEM of n=3 experiments. Legend for panel **A)** applies to **B). C)** Photographs of pigmented LM-MEL-28 spheroid cultures after 168 hrs treatment with vehicle or 25 μM monepantel. All evidence of pigmentation is lost as the cells die in culture following treatment.

Supplementary Figure 5: **LC3B-II/LC3B-I ratio increases upon monepantel treatment. A-F)** Densitometry analysis of bands corresponding to LC3-I and LC3-II from Figures 3A-C in the main text. Results are presented as the ratio of LC3B-II:LC3B-I (%).

Supplementary Figure 6: **Effect of monepantel in cell lines deficient for ATG7.** Analysis of the proportion of live, apoptotic (early, intermediate, late) and necrotic cell populations (as distinguished in Supplementary Figure 3) for **A)** OVCAR-3 and **B)** MEFs (WT or ATG7-deficient) following 24, 48, 120 hrs treatment with indicated concentrations of monepantel (MPL) or vehicle. Data is the mean±SEM of n=3 experiments.

Supplementary Figure 7: **Transcriptomics analysis of four cell lines following monepantel treatment. A)** Multi-dimensional scaling (MDS) plots comparing the indicated cell lines following monepantel treatment. **B)** Barcode plot showing enrichment of the cell cycle pathway in the comparison of monepantel vs vehicle for the indicated cell lines. Results of Roast statistical testing of the enrichment are shown under the plot.

Supp. Fig. 1

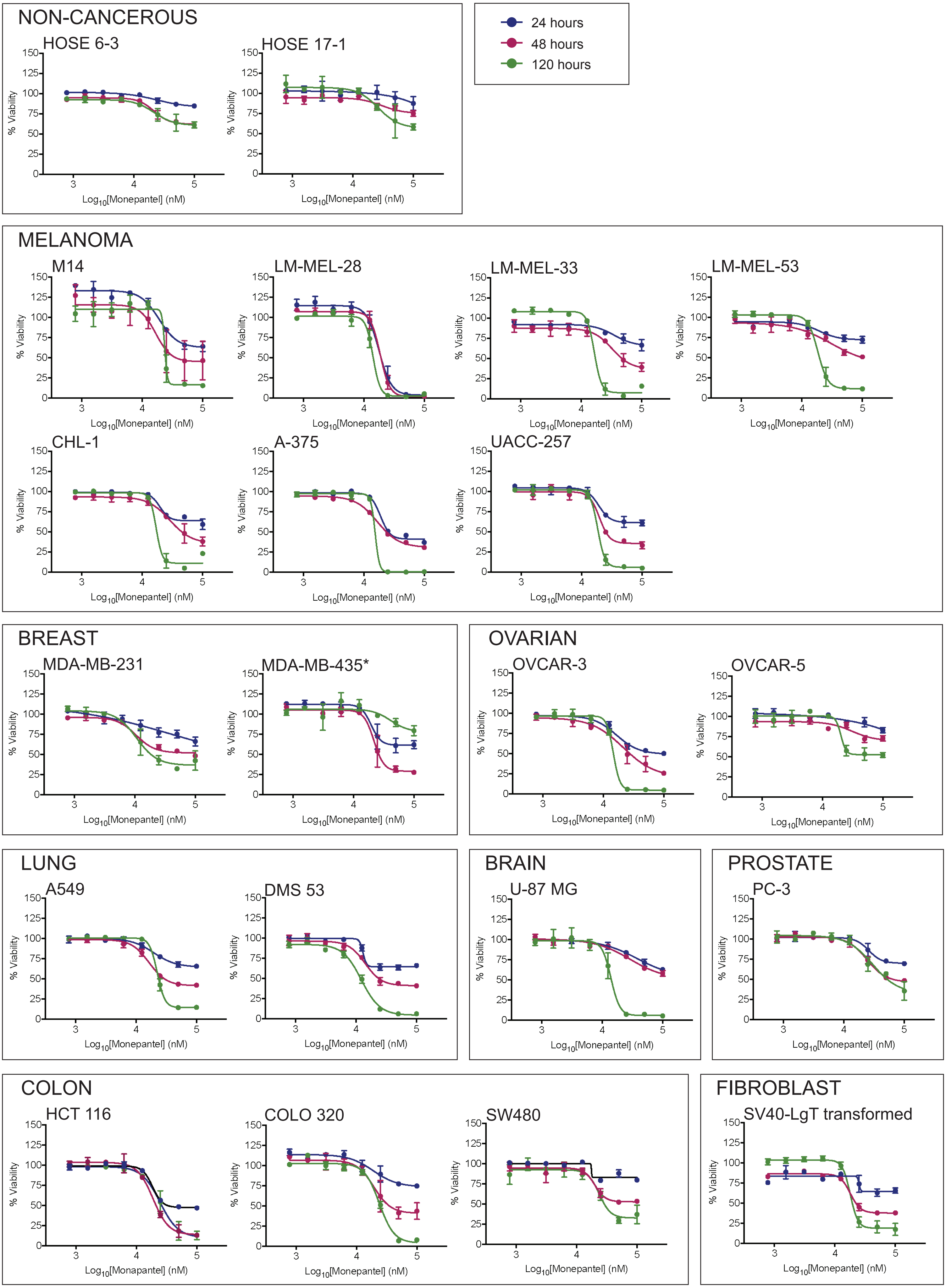
NON-CANCEROUS
HOSE 6-3
HOSE 17-1
24 hours
48 hours
120 hours
MELANOMA
M14
LM-MEL-28
LM-MEL-33
LM-MEL-53
CHL-1
A-375
UACC-257
BREAST
MDA-MB-231
MDA-MB-435*
OVARIAN
OVCAR-3
OVCAR-5
LUNG
A549
DMS 53
BRAIN
U-87 MG
PROSTATE
PC-3
COLON
HCT 116
COLO 320
SW480
FIBROBLAST
SV40-LgT transformed
% Viability
Log10[Monepantel] (nM)
Log10[Monapantel] (nM)

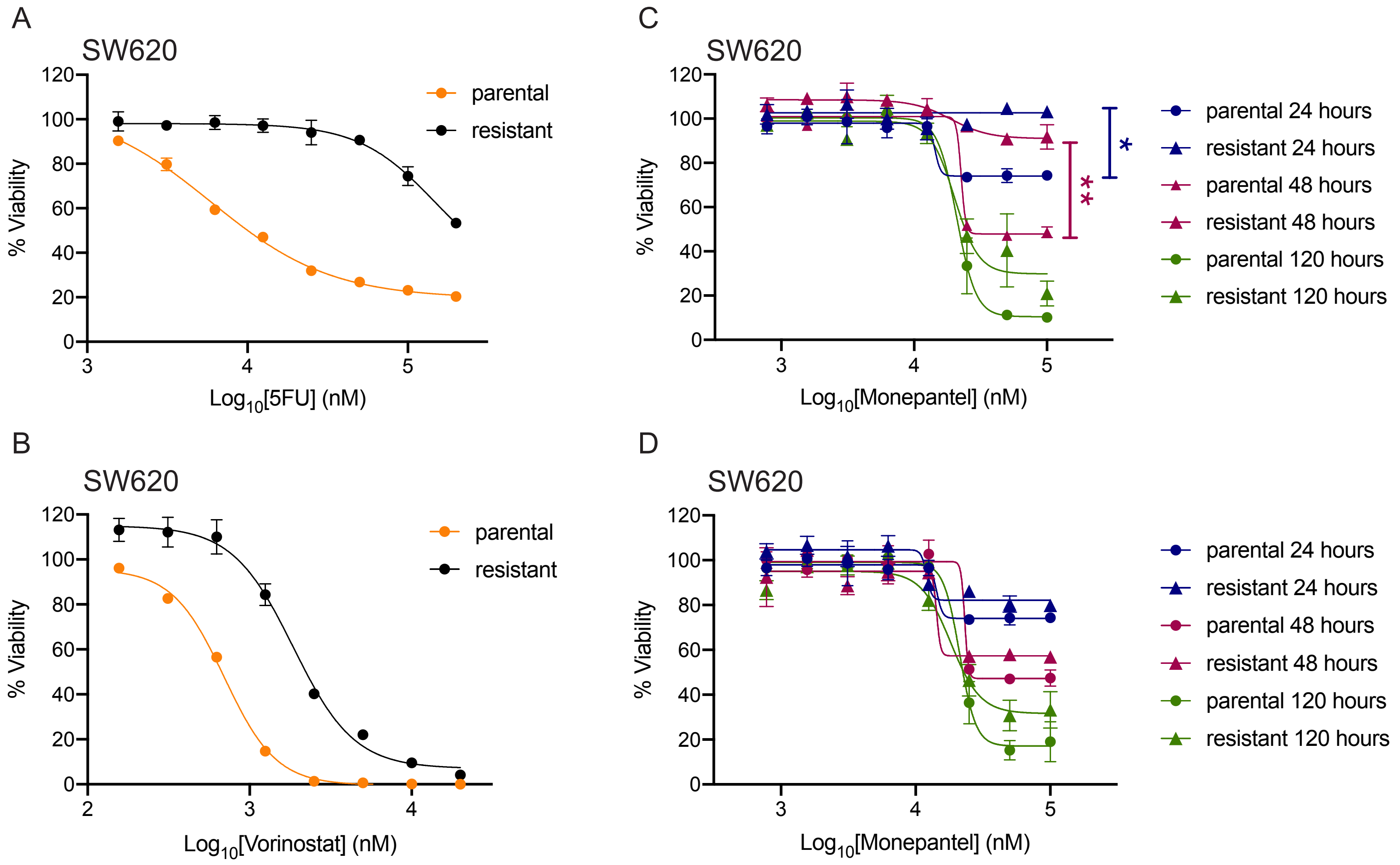
A
SW620
% Viability
Log10[5FU] (nM)
parental
resistant
B
SW620
% Viability
Log10[Vorinostat] (nM)
parental
resistant
C
SW620
% Viability
Log10[Monepantel] (nM)
parental 24 hours
resistant 24 hours
parental 48 hours
resistant 48 hours
parental 120 hours
resistant 120 hours
*
**
D
SW620
% Viability
Log10[Monepantel] (nM)
parental 24 hours
resistant 24 hours
parental 48 hours
resistant 48 hours
parental 120 hours
resistant 120 hours

Supp. Fig. 3

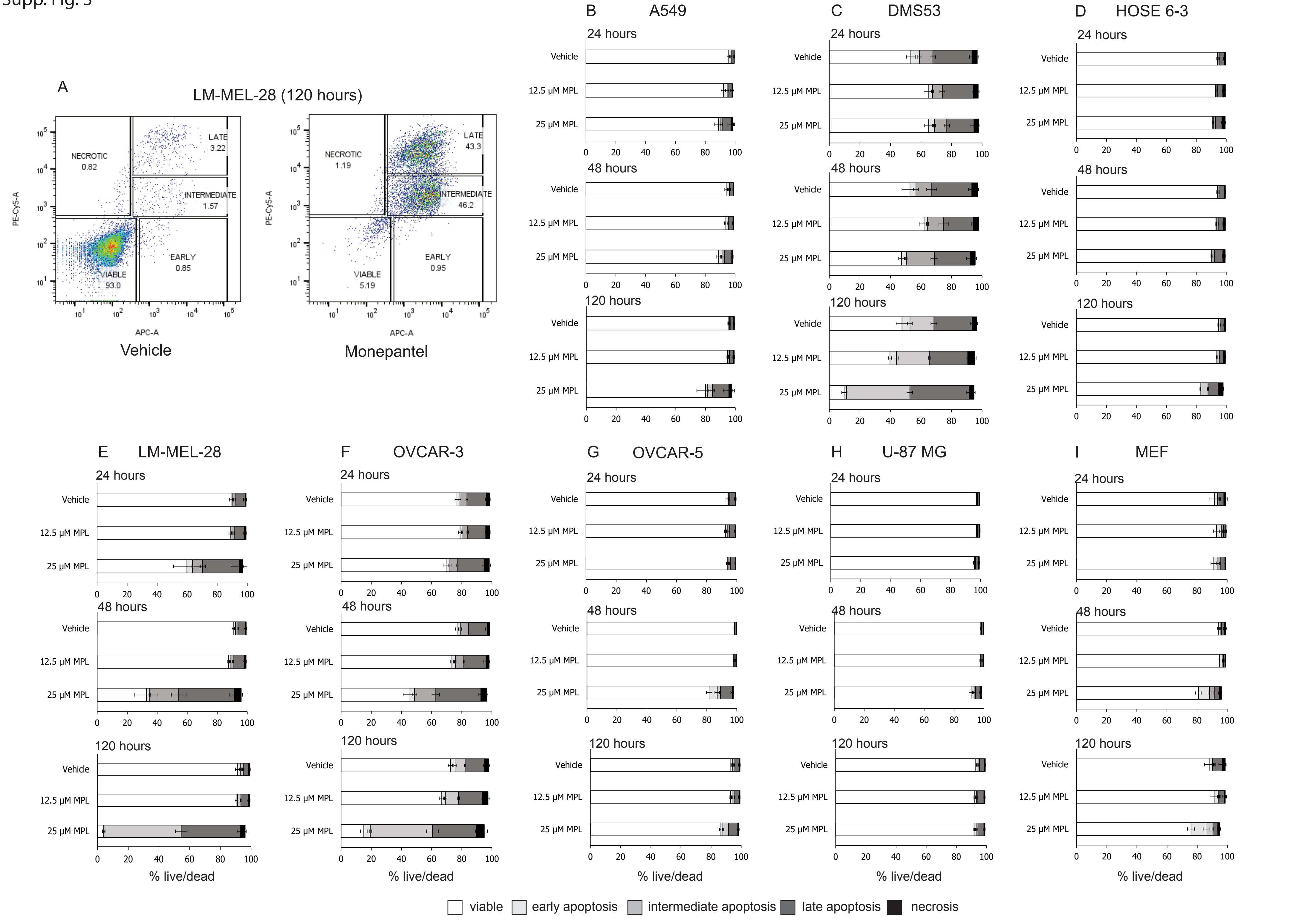

A
LM-MEL-28 (120 hours)
NECROTIC 0.82
LATE 3.22
INTERMEDIATE 1.57
EARLY 0.85
VIABLE 93.0
PE-Cy5-A
APC-A
Vehicle
NECROTIC 1.19
LATE 43.3
INTERMEDIATE 46.2
EARLY 0.95
VIABLE 5.19
Monepantel
B A549
C DMS53
D HOSE 6-3
E LM-MEL-28
F OVCAR-3
G OVCAR-5
H U-87 MG
I MEF
24 hours
48 hours
120 hours
Vehicle
12.5 µM MPL
25 µM MPL
0 20 40 60 80 100
% live/dead
viable
early apoptosis
intermediate apoptosis
late apoptosis
necrosis

A

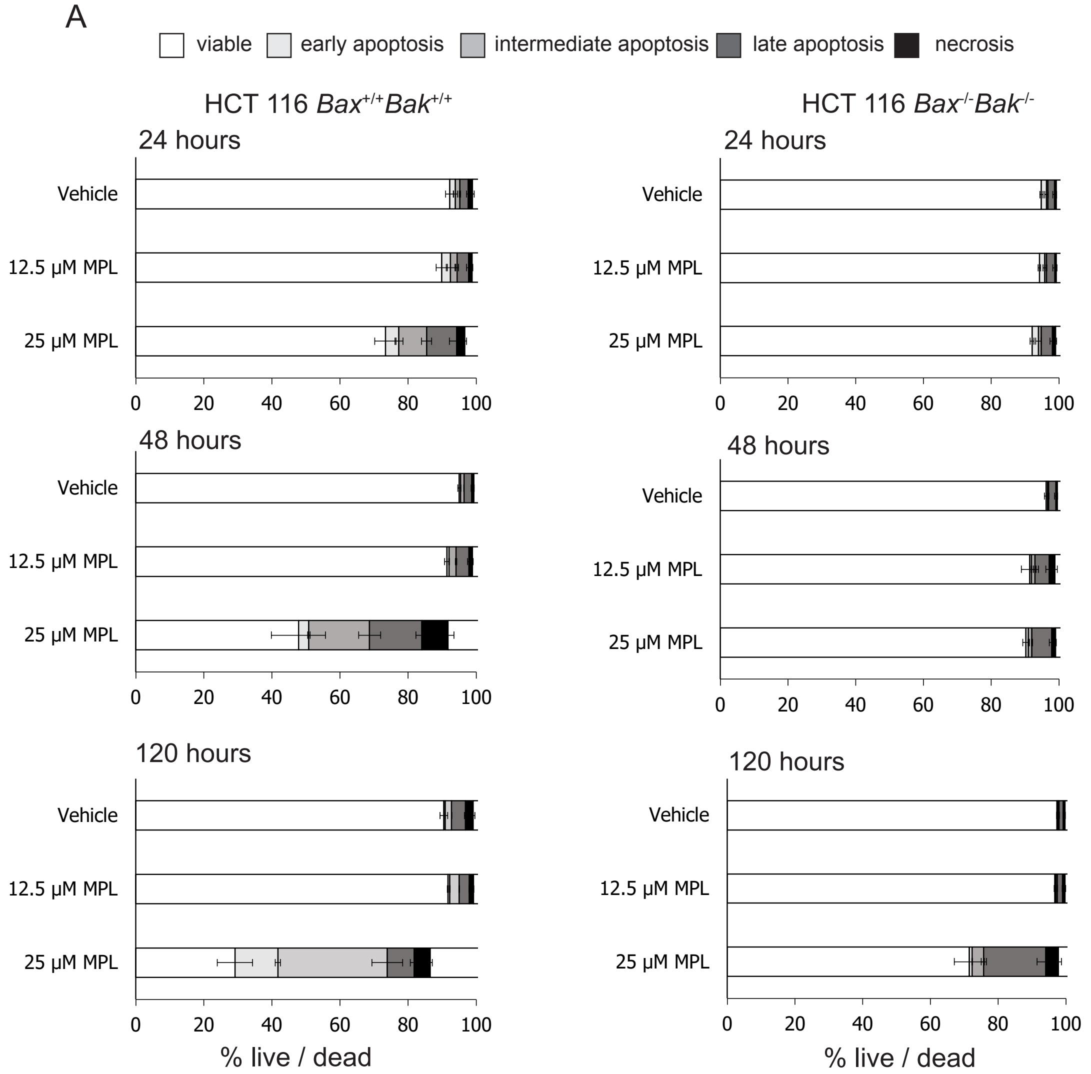

B

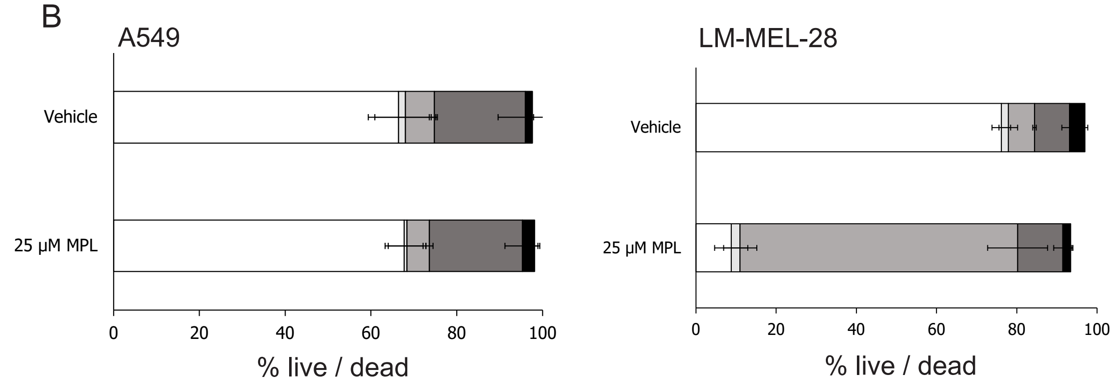

C

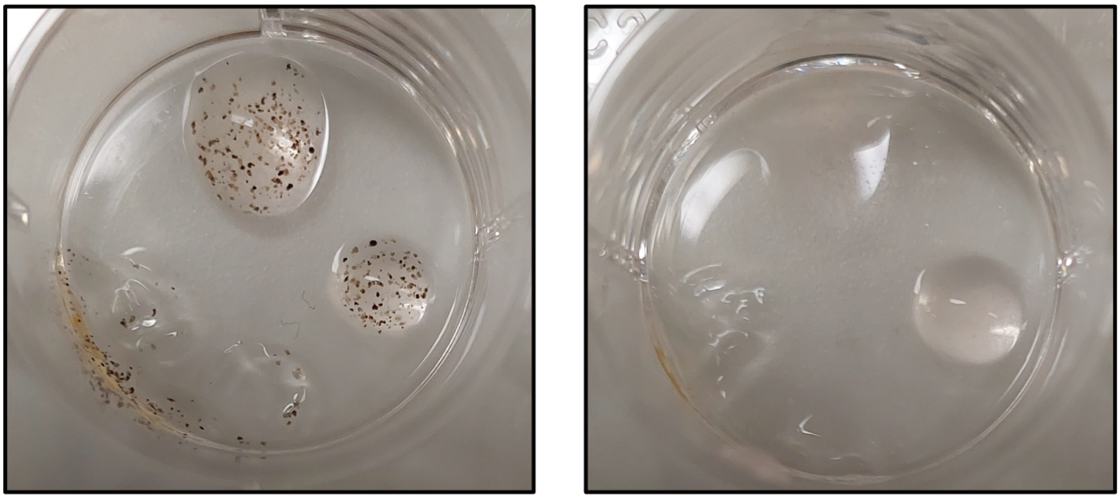

Vehicle

Monepantel

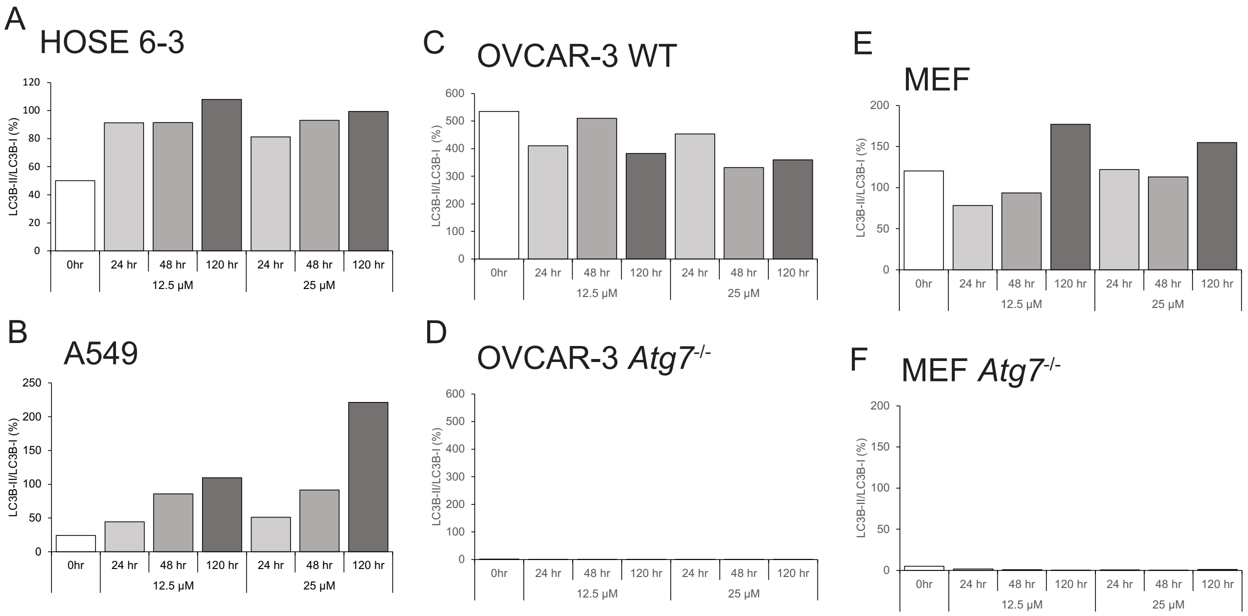
A
HOSE 6-3
LC3B-II/LC3B-I (%)
0hr
24 hr
48 hr
120 hr
12.5 µM
25 µM
B
A549
C
OVCAR-3 WT
D
OVCAR-3 Atg7-/-
E
MEF
F
MEF Atg7-/-

Supp. Fig. 6

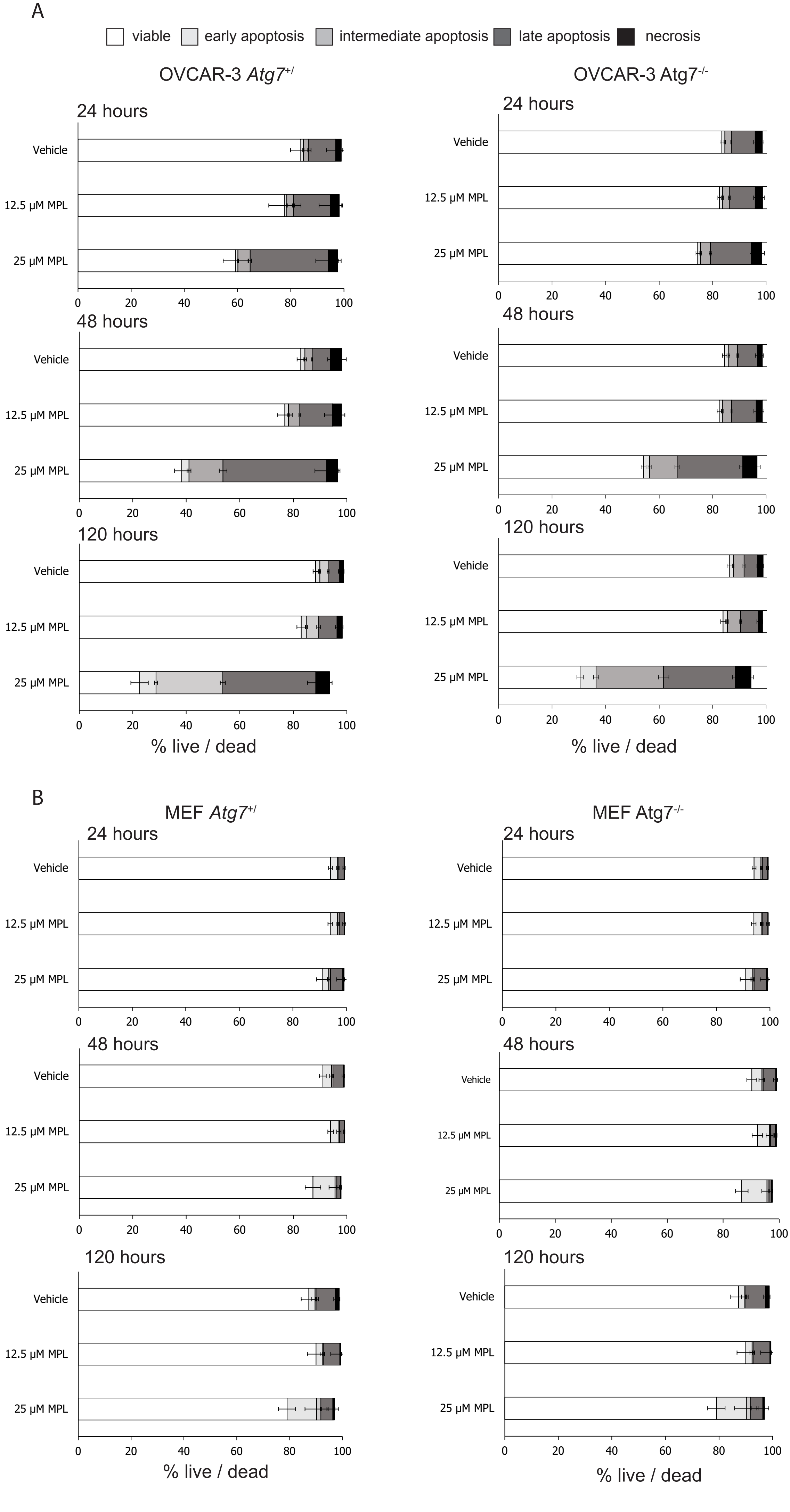
A
viable
early apoptosis
intermediate apoptosis
late apoptosis
necrosis
OVCAR-3 *Atg7*+/
OVCAR-3 Atg7-/-
24 hours
48 hours
120 hours
Vehicle
12.5 µM MPL
25 µM MPL
0
20
40
60
80
100
% live / dead
B
MEF *Atg7*+/
MEF Atg7-/-

Supp Fig. 7

A

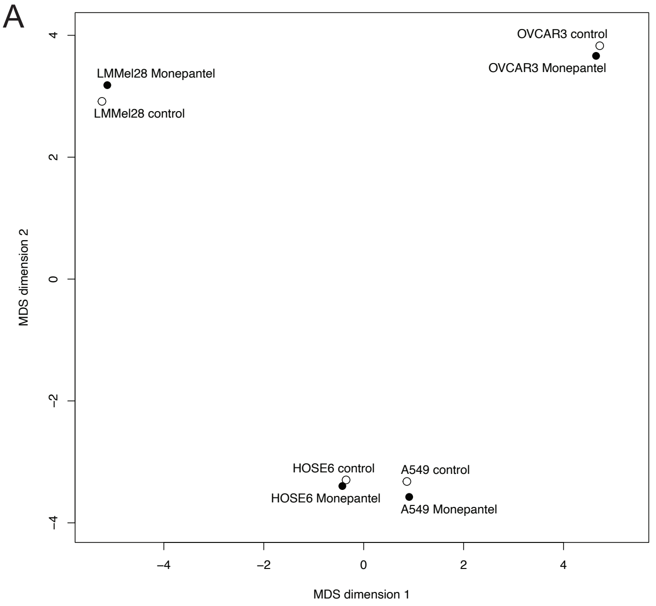

B

HOSE 6-3

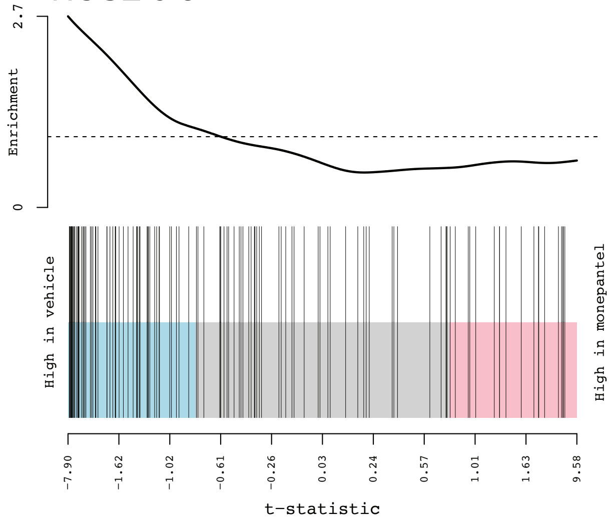

	Up	Down	UpOrDown	Mixed
Act. Prop.	0.0813	0.3496	0.3496	0.4309
P. value	0.9969	0.0032	0.0064	0.0073

A549

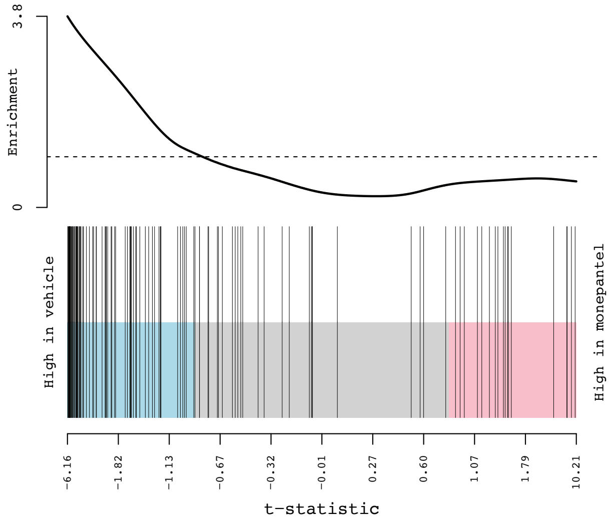

	Up	Down	UpOrDown	Mixed
Act. Prop.	0.0732	0.5285	0.5285	0.6016
P. value	1.0000	0.0000	0.0001	0.0002

OVCAR-3

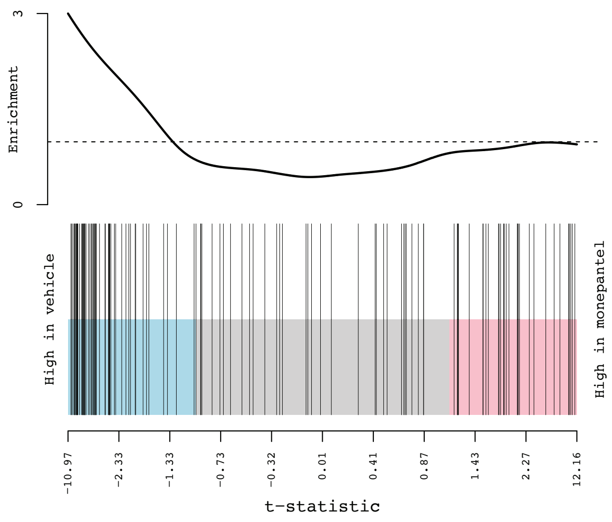

	Up	Down	UpOrDown	Mixed
Act. Prop.	0.2033	0.4553	0.4553	0.6585
P. value	0.9995	0.0005	0.0011	0.0003

LM-MEL-28

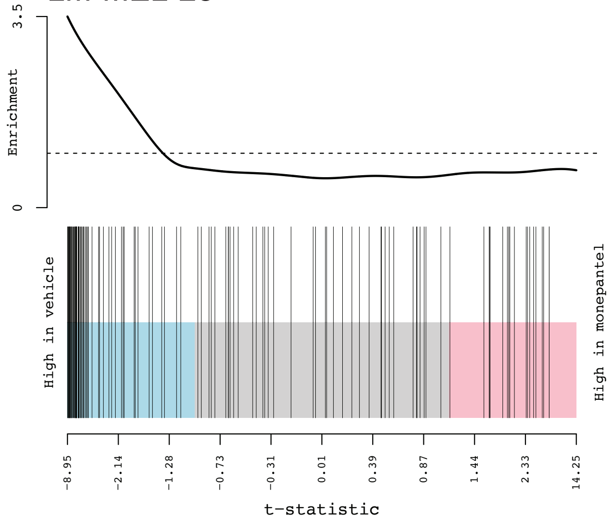

	Up	Down	UpOrDown	Mixed
Act. Prop.	0.1463	0.4959	0.4959	0.6423
P. value	1.0000	0.0000	0.0001	0.0001
